# Gut microbiota-mediated activation of GSDMD ignites colorectal tumorigenesis

**DOI:** 10.1038/s41417-024-00796-2

**Published:** 2024-06-19

**Authors:** Ju Chen, Neha Singh, Xiaoyang Ye, Eileen Victoria Theune, Kepeng Wang

**Affiliations:** 1 Department of Immunology, School of Medicine, University of Connecticut Health Center, 263 Farmington Ave., Farmington, CT 06030 USA; 2 The Eighth Clinical Medical College of Guangzhou University of Chinese Medicine, Foshan Hospital of Traditional Chinese Medicine, Foshan, Guangdong 528000 China

**Keywords:** Colorectal cancer, Innate immunity

## Abstract

Activation of Gasdermin D (GSDMD) results in its cleavage, oligomerization, and subsequent formation of plasma membrane pores, leading to a form of inflammatory cell death denoted as pyroptosis. The roles of GSDMD in inflammation and immune responses to infection are well documented. However, whether GSDMD also plays a role in sporadic cancer development, especially that in the gut epithelium, remains unknown. Here, we show that GSDMD is activated in colorectal tumors of both human and mouse origins. Ablation of GSDMD in a mouse model of sporadic colorectal cancer resulted in reduced tumor formation in the colon and rectum, suggesting a tumor-promoting role of the protein in the gut. Both antibiotic-mediated depletion of gut microbiota and pharmacological inhibition of NLRP3 inflammasome reduced the activation of GSDMD. Loss of GSDMD resulted in reduced infiltration of immature myeloid cells, and increased numbers of macrophages in colorectal tumors. Activation of GSDMD is also accompanied by the aggregation of the endosomal sorting complex required for transport (ESCRT) membrane repair proteins on the membrane of colorectal tumor cells, suggesting that active membrane repairment may prevent pyroptosis induced by the formation of GSDMD pore in tumor cells. Our results show that gut microbiota/NLRP3-mediated activation of GSDMD promotes the development of colorectal tumors, and supports the use of NLRP3 inhibitors to treat colon cancer.

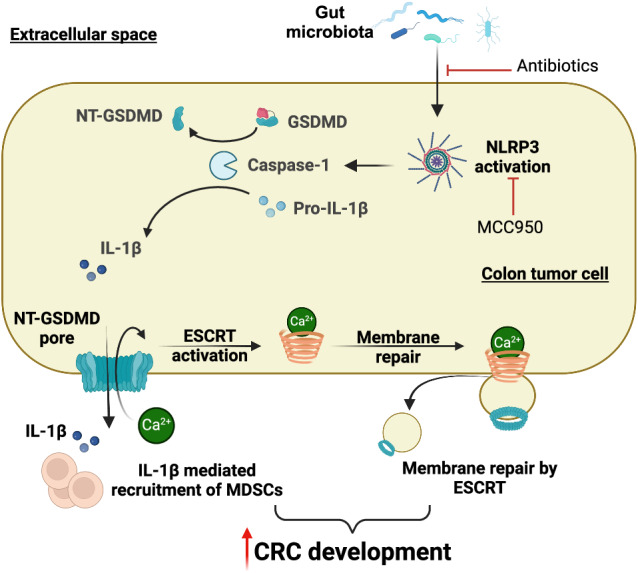

## Introduction

Colorectal cancer (CRC) is the third leading cause of cancer-related deaths in the United States [1]. Recent studies have illustrated critical roles for tumor-associated inflammation in the development of CRC and other solid tumors [2–5]. A common paradigm of these inflammatory processes is their support of tumor cell survival and proliferation, and the remodeling of the tumor immune environment to one that favors the evasion of tumor cells from immune destruction [2–5]. In CRC, tumor-associated inflammation is sponsored by the loss of epithelial surface barrier function upon gut epithelium transformation, followed by the infiltration of commensal bacteria and their degradative products to tumor stroma [6]. This “tumor elicited inflammation” is characterized by the infiltration of myeloid and Th17 cells to colorectal tumors, and the production of cytokines such as IL-23 and IL-17 that support tumor cell proliferation and survival [6, 7].

Inflammasomes are intracellular innate immune structures that play a role in host defense against bacteria, inflammation, and cancer [8, 9]. Under stress conditions, the activation of pattern recognition receptor (PRR) signaling initiates a response to microbial components through sensing pathogen-associated molecular patterns (PAMPs), and several components derived from damaged or dying cells (damage-associated molecular patterns, DAMPs) [10]. Among the PRRs are the nucleotide-binding oligomerization domain (NOD), leucine-rich repeat (LRR)-containing protein (NLR) family members, including NLRP3, NLRC4, AIM2, and others [11]. These receptors recognize a broad spectrum of PAMPs and DAMPs that can trigger the cleavage of pro-caspase 1 into its mature form, which in turn leads to the cleavage of IL-1 family cytokines IL-1β and IL-18, as well as cleavage of Gasdermin D (GSDMD) [12–15]. Importantly, the N-terminus of GSDMD subsequently oligomerizes and forms pores on the plasma membrane, resulting in the release of mature IL-1β and IL-18, as well as pyroptosis [16–18]. GSDMD also drives pyroptosis through the “non-canonical” inflammasome pathway, following the activation of caspase 11 in mice and caspase 4 and 5 in humans [19, 20]. The formation of GSDMD membrane pores does not necessarily lead to pyroptosis, as membrane pore formation also activates the Ca^2+^-dependent endosomal sorting complex required for transport (ESCRT). ESCRT activation initiates membrane repair which can maintain cell viability while allowing prolonged IL-1 family cytokine release [21].

Studies of inflammasome receptors and GSDMD have shed light on their roles in cancers. GSDMD in myeloid cells contributes to the lung metastasis of Lewis lung carcinoma (LLC) cells [22]. GSDMD was also found to be over-expressed in non-small cell lung cancer (NSCLC), and its ablation enhanced the rate of cancer cell apoptosis [23]. GSDMD was found to repress tumor development in a mouse model of colitis-associated colon cancer, which was attributed to its role in protecting mice from DSS-induced colitis [24]. Protein mediators involved in the multiple inflammasome pathways that activate GSDMD, including NLRP1, NLRP3, and AIM2, have been shown to attenuate the development of colitis-associated colorectal cancer (CAC) in mice [25–28]. The anti-tumor function of these three inflammasomes also relies on their protection from colitis, which is required for tumor induction in the AOM/DSS model of CAC. However, NLRP3 was also found to promote CAC development in mice treated with high cholesterol diet [29]. Activation of NLRP3 and release of IL-1β was also reported to cause overproduction of 5-hydroxytryptamine in colorectal tumors, which accelerates CAC development [30]. CAC accounts for less than 5% of total CRC in humans, and about 80% of human CRC originates from the aberrant activation of the Wnt/β-catenin pathway, which is modeled in the current study.

To date, it is not clear if inflammasome signaling activates GSDMD in sporadic CRC, the major form of CRC in humans. It is also unknown if inflammasome/GSDMD signaling is activated in the tumor, and if so, in which cells is the pathway activated, and how does such activation impacts the fate of these cells. We employed two mouse models of CRC to fill this major gap: a sporadic CRC model that depends on the spontaneous loss of heterozygosity of the *Apc* tumor suppressor, and an inducible CRC model that allows the study of early-phase CRC development. We ablated GSDMD in the whole body and in hematopoietic cells in these CRC models, and now report a tumor-promoting role of GSDMD in sporadic CRC that is dependent on NLRP3 and gut bacteria.

## Materials and methods

### Animal models

C57BL/6, *Apc*^*F/F*^, *Cdx2-Cre*, and *Cdx2-Cre-ERT2* mice were obtained from the Jackson Laboratory. *Gsdmd*^−/−^ mice were obtained from Genentech [31]. Sample size for each experiment was estimated based on previously reported preclinical studies [32]. To generate the mouse model of sporadic CRC, *Cdx2-Cre* and *Apc*^*F/F*^ mice were crossed to generate *Cdx2-Cre*^*+*^/*Apc*^*F/+*^ mice. These mice were sacrificed around 5 months of age for tumor analyses. Mouse colon was dissected, and colorectal tumors were measured with a caliper. Tumors were then excised with a scissor. For comparison, tumor-adjacent colon tissues were harvested and analyzed as “normal colon tissue”.

For tamoxifen-inducible tumorigenesis, *Cdx2-Cre-ERT2*^*+*^*/Apc*^*F/F*^ mice were given *i.p*. injections of 75 mg/kg body weight tamoxifen (Sigma, dissolved in 5% ethanol, 95% corn oil) daily for 3 consecutive days. Mice were sacrificed 4–5 weeks after the last dose of tamoxifen. Mouse colon was dissected, and visible colorectal tumors (typically 1–2 mm in diameter) were measured with a caliper and excised with a scissor for analyses.

For antibiotic treatment of mice, mice were given 100 μg/ml neomycin, 50 μg/ml vancomycin, 50 μg/ml imipenem, 100 μg/ml metronidazole, 50 μg/ml streptomycin and 100 units/ml penicillin in drinking water for 3 weeks. Fresh antibiotic water was supplied every week.

For NLRP3 inhibitor treatment of mice, 5-month-old *Cdx2-Cre*^*+*^/*Apc*^F/+^ mice bearing colorectal tumors were given *i.p*. injection of NLRP3 inhibitor MCC950 (10 mg/kg body weight) once every 2 days. A total of 4 doses were given, and mice were sacrificed one day after the last dose for analysis.

For animal experiments, none of the animals were excluded from the study. Animals were randomly assigned during treatments. Blinding was performed during data analysis. All mice were maintained in specific-pathogen-free conditions with filter-topped cages on autoclaved food and water at UConn Health, and all experiments were approved by the IACUC of UConn Health and performed in accordance with the university and NIH guidelines and regulations. All experiments used co-housed, gender-matched littermates to ensure the consistency of common microflora.

### Bone marrow transplantation

Six- to eight-week-old recipient mice received lethal dose (900 rad) irradiation and intravenous injection with single-cell suspension of 10^7^ donor bone marrow cells. Recipient mice were then supplied with sulphamethoxazole and trimethoprime in drinking water for two weeks, followed by regular water. Recipients were co-housed littermates, which were transplanted with both gene-deficient and wild-type bone marrow for comparison. Mice of the sporadic CRC model (*Cdx2-Cre*^*+*^/*Apc*^*F/+*^) were sacrificed and analyzed for tumor development 3–4 months after transplantation. For the inducible CRC model (*Cdx2-Cre-ERT2*^*+*^*/Apc*^*F/F*^), recipient mice were also given tamoxifen injection at least two months after bone marrow transfer.

### Flow cytometry

Colorectal tumors were minced with scissors and digested with 1 mg/ml collagenase IV (Sigma Aldrich, Cat # C5138) for 20 min. Cells were filtered with a 70-μm cell sieve and stained with Live/Dead fixable exclusion dye (Tonbo Bioscience, Cat # 13-0868), followed by fluorochrome-conjugated antibodies in PBS with 2% fetal bovine serum (FBS) and 1 mM EDTA. Anti-CD3 (Cat # 100206), anti-CD4 (Cat # 100536), anti-CD45 (Cat # 103138), anti-CD11b (Cat # 101224), anti-F4/80 (Cat # 123108), anti-Gr-1 (Cat # 108428), anti-Ly6C (Cat # 128016), anti-Ly6G (Cat # 127641), and anti-IL-17A (Cat # 506904) antibodies were from Biolegend. Anti-Foxp3 (Cat # 11-5773-82) antibody was from eBioscience. For intracellular cytokine staining, cells were restimulated in the presence of PMA, Ionomycin, and Brefeldin A for 4 h, stained with Live/Dead fixable dye and surface antigen targeting antibodies, followed by fixation and staining with labeled antibodies for the cytokine of interest. Fixation and permeabilization of cells were done with the Foxp3/transcription factor staining buffer set (eBioscience, Cat # 00-5523-00) according to the manufacturer’s recommendations. Flow cytometry analyses were performed on a BD LSRII flow cytometer. Data were analyzed using FlowJo software.

### Immunoblotting analysis

Normal colons and colorectal tumors from sporadic and inducible CRC mouse models were minced with scissors and lysed in RIPA buffer with protease and phosphatase inhibitor cocktail (ThermoFisher, Cat # 78440). Cell lysates were centrifugated at 4 °C, 20,000 × *g* for 15 min, and total proteins in the supernatant were separated by standard SDS-PAGE and analyzed by Western blotting. α-Tubulin (Abcam, Cat # ab24246) and full-length GSDMD (Abcam, Cat # ab219800) antibodies were used in this study. NLRP3, AIM2, ASC, Cleaved-Caspase 1, Caspase 11, and IL-1β antibodies were from the Mouse Reactive Inflammasome Antibody Sampler Kit (Cell Signaling Technology, Cat # 20836). Additional antibodies include IL-1α (Cell Signaling Technology, Cat # 50794 S), IL-1β (Sigma Aldrich, Cat # I3767), cleaved IL-1β (Cell Signaling Technology, Cat # 63124) and IL-18 (BioVision, Cat # 5180R).

### Immunofluorescence staining

For cell surface antigen staining, human or mouse colonic tumor tissue slides were fixed using −20 °C Methanol/acetone (1:1) for 3 min, followed by washing with PBS three times. Then specimens were blocked using a blocking buffer (1% BSA, 1% donkey serum in PBS) and incubated with primary antibody in blocking buffer at room temperature (RT) for 1 h. After washing twice with the blocking buffer and once with PBS, slides were fixed using 1% PFA for 10 min, followed by three washes with PBS and blocked with permeable blocking buffer (1% BSA, 1% Donkey serum, 0.3% Triton in PBS) at RT for 1 h. Specimens were then incubated in permeable blocking buffer with the primary antibodies targeting intracellular antigens at 4 °C overnight in a humid chamber, washed three times with PBS, followed by incubation in the dark with secondary antibodies at RT for 1 h. After being washed twice by PBS with 1% BSA, and 0.3% Triton, slides were embedded in Vector Mounting Medium with DAPI in the dark at RT for 10 min. Slides were observed using a laser-scanning confocal microscope (ZEISS, LSM 880 confocal). Anti-NLRP3 and anti-ASC antibodies were from Cell Signaling Technology (Cat # 20836). Anti-CHMP2A and anti-CHMP4B were from Proteintech Group Inc.

### Real-time PCR and gene expression analysis

Total RNA was isolated from tumor samples using the RNeasy mini kit (Qiagen, Cat # 74134). 1 μg total RNA was reverse transcribed by using i-Script cDNA Synthesis System kit (Bio-Rad, Cat # 1708891) following the manufacturer’s protocol. To measure gene expression, real-time PCR was performed using SsoAdvanced™ Universal SYBR® Green Supermix (Bio-Rad, Cat # 1725271), following the manufacturer’s protocol. Expression levels of each transcript were quantified by using Bio-Rad CFX96 Real-Time PCR Detection System. GAPDH was used as an endogenous control.

Primer sequences are as following:

Mouse IL-1β: Forward primer 5′ GCCACCTTTTGACAGTGATGAG 3′; Reverse primer 5′ GACAGCCCAGGTCAAAGGTT 3′, Amplicon = 95

Mouse IL-1α: Forward primer 5′ AGGGAGTCAACTCATTGGCG 3′; Reverse primer 5′ TGGCAGAACTGTAGTCTTCGT 3′, Amplicon = 116

Mouse IL-18: Forward primer 5′ GGCTGCCATGTCAGAAGACT 3′; Reverse primer 5′ ACAGTGAAGTCGGCCAAAGT 3′, Amplicon = 24

GAPDH: Forward primer 5′ TGGTGAAGGTCGGTGTGAAC 3′; Reverse primer 5′ GAAGGGGTCGTTGATGGCAA 3′, Amplicon = 104

### Statistical analysis

Data were analyzed by the Students’ *t*-test for pair-wise comparisons without assuming equal variance, and one-way ANOVA for comparison between three or more groups. *p* values less than 0.05 were considered significant.

## Results

### GSDMD is activated in multiple cancers

**Fig. 1:**
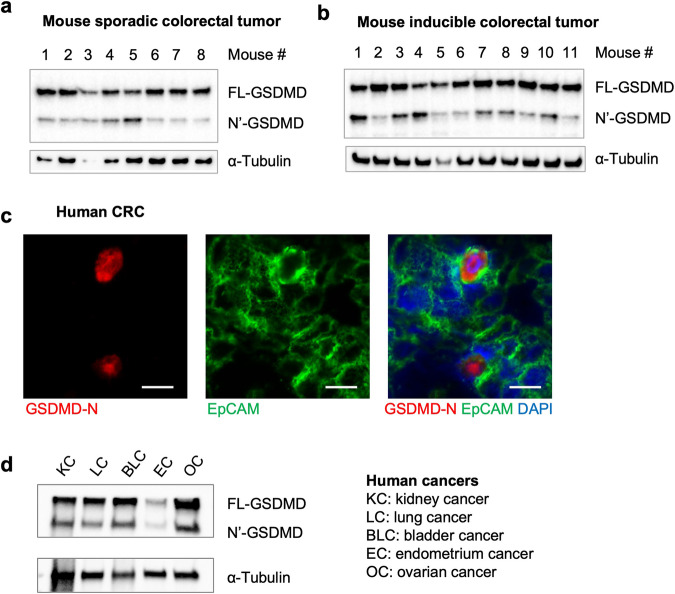
Cleavage of GSDMD in multiple cancers. **a** 5-month-old *Cdx2-Cre*^*+*^/*Apc*^F/+^ mice (sporadic CRC model) bearing colorectal tumors were sacrificed, and their tumors were analyzed by Western blotting using an antibody that recognizes both the full-length form (FL) and N’-terminus (N’) of GSDMD. **b** 2-month-old *Cdx2-Cre-ERT2*^*+*^/*Apc*^F/F^ mice (inducible CRC model) were given three daily *i.p*. injections of 70 mg/kg tamoxifen to ablate both copies of *Apc*. Mice were sacrificed 1 month after the last tamoxifen injection, and colonic tumors were analyzed by Western blotting. **c** Human colorectal adenocarcinoma specimens were cryosectioned and stained with antibodies against cleaved GSDMD, Ep-CAM, and DAPI. Images are representative of 4 human CRC cases. **d** Human cancers of the kidney (KC), lung (LC), bladder (BLC), endometrium (EC), and ovary (OC) were analyzed by Western blotting to detect full-length (FL) and N’-terminus (N’) GSDMD. α-Tubulin blotting was used as a loading control.

We employed two mouse models of CRC to interrogate the involvement of GSDMD in cancer. The mouse model of sporadic CRC (*Cdx2-Cre*^*+*^/*Apc*^F/+^ mice) is based on the allelic inactivation of one copy of the *Apc* tumor suppressor gene in colonic epithelial cells, driven by a *Cdx2-Cre* transgene [33, 34]. Subsequent *Apc* loss-of-heterozygosity results in the development of large adenomas and adenocarcinomas in the large intestine [34]. Tumors in this model are restricted to the colon and rectum, and progress to adenocarcinomas, which makes this model very relevant to human CRC. We also used a second mouse model of synchronized tumorigenesis in the colon that relies on tamoxifen-induced ablation of the *Apc* gene (*Cdx2-Cre-ERT2*^*+*^*/Apc*^F/F^ mice), and allows for the study of early-stage colorectal tumors [35]. Early CRC lesions can be detected by histology 1 week after tamoxifen injection, and progress to visible colorectal tumors by 4 weeks. In both models, we observed robust cleavage of GSDMD in colorectal tumors, suggesting a potential involvement of GSDMD in CRC pathogenesis (Fig. 1a, b). When stained with an antibody that detects cleaved (thus activated) human GSDMD, human CRC tissues exhibited GSDMD activation mostly in transformed epithelial cells (Fig. 1c). GSDMD activation was also observed in human cancers of the kidney, lung, bladder, uterus, and ovary (Fig. 1d). These observations suggest that GSDMD may play a role in multiple cancers, including cancers in the gut.

### GSDMD in radio-resistant cells promotes colorectal tumorigenesis

**Fig. 2:**
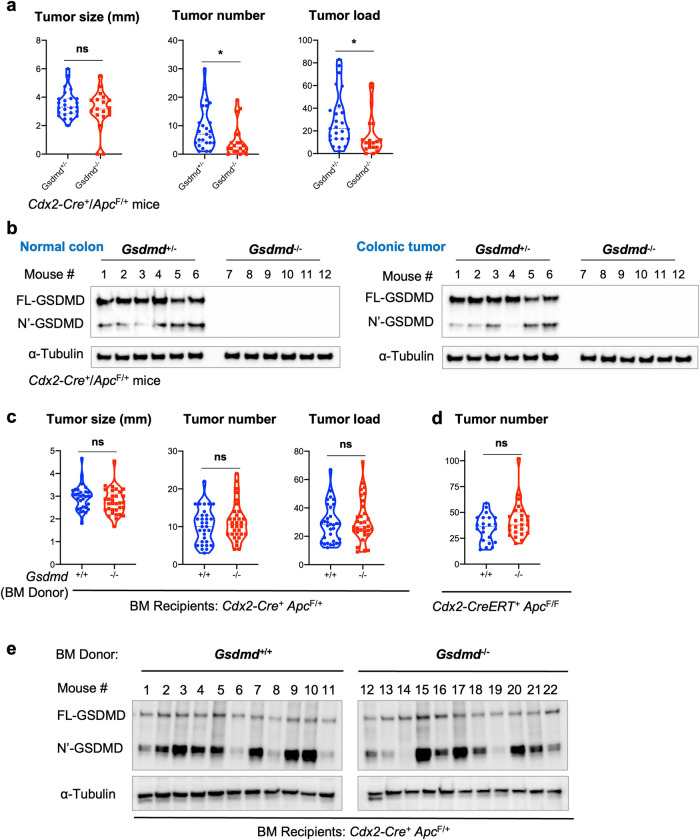
GSDMD in radiation-resistant cells promotes CRC development. **a** 5-month-old *Cdx2-Cre*^*+*^/*Apc*^F/+^ mice that harbor heterozygous (*Gsdmd*^+/−^, used as controls) or null (*Gsdmd*^−/−^) alleles of GSDMD were sacrificed and subjected to colorectal tumor statistics. Tumor load was calculated as the summation of tumor diameters for each mouse, *n* = 19. **b** Normal colon tissue and colorectal tumors from 5-month-old *Cdx2-Cre*^*+*^/*Apc*^F/+^ mice harboring *Gsdmd*^+/−^ or *Gsdmd*^−/−^ alleles were dissected and analyzed by Western blotting. **c**, **d** Bone marrow cells were harvested from *Gsdmd*^+/+^ and *Gsdmd*^−/−^ mice, and transferred into lethally irradiated 6–8-week-old *Cdx2-Cre*^+^/*Apc*^F/+^ (*n* = 31) mice (**c**) or *Cdx2-Cre-ERT2*^*+*^/*Apc*^F/F^ (*n* = 22) mice (**d**). **c** *Cdx2-Cre*^+^/*Apc*^F/+^ mice were sacrificed at 5 months of age for tumor statistics. **d** *Cdx2-Cre-ERT2*^*+*^/*Apc*^F/F^ mice received 3 doses of 75 mg/kg tamoxifen 2 months following bone marrow transfer and were sacrificed 1 month later. The numbers of visible colonic tumors were counted and shown. **e** 6–8-week-old *Cdx2-Cre*^+^/*Apc*^F/+^ mice received bone marrow cell transfer from *Gsdmd*^+/+^ or *Gsdmd*^−/−^ donors, and were sacrificed at 5 months of age. Their colorectal tumors were dissected and analyzed by Western blotting. Each lane represents the pooled tumor lysate of one tumor-bearing mouse. *p < 0.05.

To interrogate the role of GSDMD in CRC, we crossed *Gsdmd*^−/−^ mice [31] to *Cdx2-Cre* and *Apc*^F/F^ mice, to ablate GSDMD in our mouse model of sporadic CRC. *Gsdmd*^−/−^ /*Cdx2-Cre*^*+*^ /*Apc*^F/+^ mice developed significantly fewer colorectal tumors compared to *Gsdmd*^+/−^/*Cdx2-Cre*^*+*^/*Apc*^F/+^ controls (Fig. 2a), demonstrating a previously unreported role of GSDMD in promoting sporadic CRC development. We probed for the full-length GSDMD and N’-GSDMD protein both in normal colon and colonic tumors, and confirmed the whole-body ablation of *Gsdmd* in sporadic CRC mice (Fig. 2b). GSDMD can be activated in several cell types, including myeloid cells, fibroblasts, and epithelial cells [36–38]. To test if GSDMD in hematopoietic cells promotes CRC, we performed an adoptive transfer of bone marrow cells from WT or *Gsdmd*^−/−^ mice to lethally irradiated *Cdx2-Cre*^*+*^/*Apc*^F/+^ recipients. Reconstitution of the hematopoietic compartment with *Gsdmd*^−/−^ cells did not change the rate of colorectal tumorigenesis (Fig. 2c). We also performed similar bone marrow transfer to the mouse model of inducible CRC (*Cdx2-Cre-ERT2*^*+*^*/Apc*^F/F^ mice). Ablation of GSDMD in hematopoietic cells also did not impact the development of early-stage colon tumors (Fig. 2d). These results indicate that GSDMD mainly functions in radio-resistant cells to promote colon cancer development. Consistent with this notion, ablation of GSDMD in hematopoietic cells did not result in an observable reduction in the full-length or cleaved GSDMD levels in the whole tumor (Fig. 2e). This also agrees with our observation that in human CRC, activation of GSDMD mainly occurs in transformed epithelial cells (Fig. 1c).

### GSDMD promotes the accumulation of immature myeloid cells in CRC

**Fig. 3:**
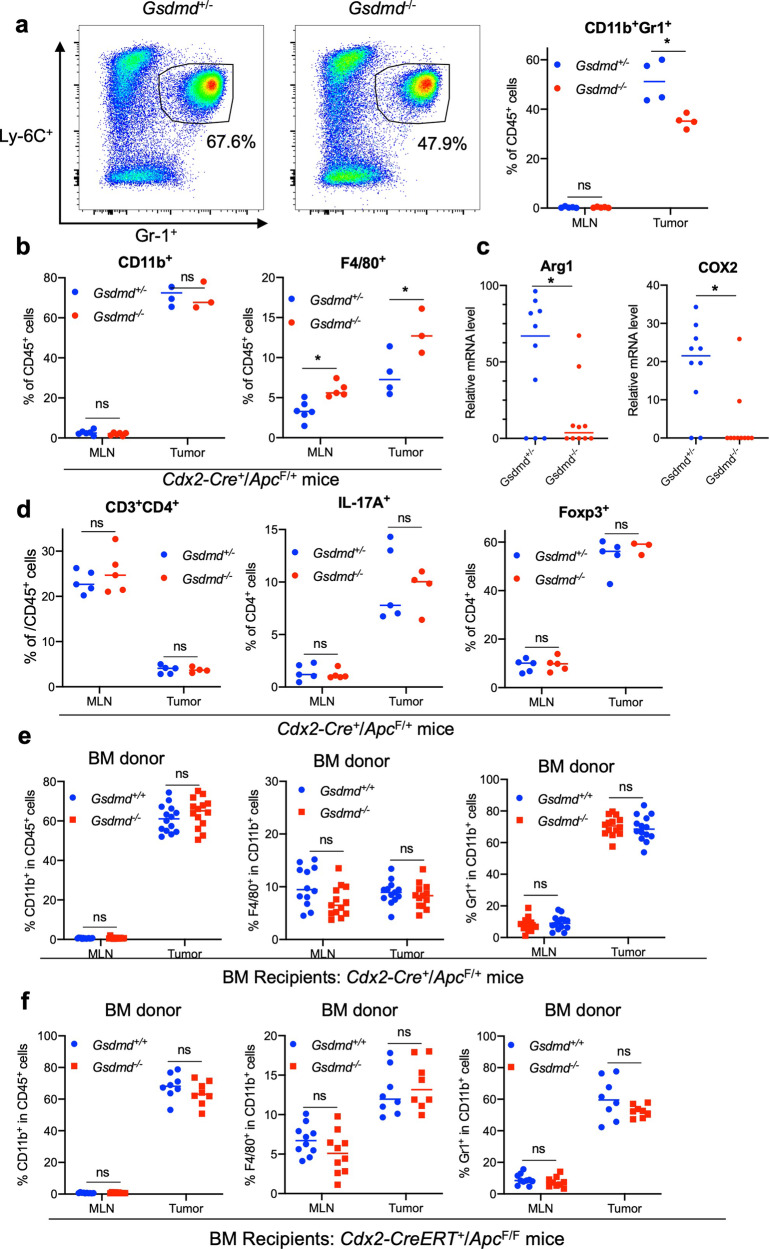
GSDMD promotes the accumulation of immature myeloid cells in colorectal tumors. **a**, **b**, **d** 5-month-old *Cdx2-Cre*^*+*^/*Apc*^F/+^ mice that harbor heterozygous (*Gsdmd*^+/−^) or null (*Gsdmd*^−/−^) alleles of GSDMD were sacrificed. Their mesenteric lymph nodes (MLN) and colorectal tumors were digested with collagenase, stained with fluorescent-conjugated antibodies and a live/dead dye, and analyzed by flow cytometry (*n* = 7). **a** Representative flow cytometry staining of Gr-1 and Ly-6C staining of dissociated colorectal tumor cells gated on live cells/CD45^+^/CD11b^+^ populations. Right panel shows the statistics of this staining. **b** Percentages of CD11b^+^ cells in live and CD45^+^ cells in MLN and colorectal tumors (left panel), and percentages of F4/80^+^ cells in CD11b^+^ population (right panel). **c** Tumors were lysed for mRNA extraction and analyzed by bulk RNA sequencing to determine the mRNA levels of arginase 1 (Arg1) and COX2 (*n* = 10). **d** Percentages of CD3^+^/CD4^+^ cells in live and CD45^+^ cells (left panel), IL-17A^+^ cells in live/CD45^+^/CD3^+^/CD4^+^ cells (middle panel), and FOXP3^+^ cells in live/CD45^+^/CD3^+^/CD4^+^ cells in MLN and colorectal tumors. **e**, **f** Bone marrow cells were harvested from WT and *Gsdmd*^−/−^ mice and transferred into lethally irradiated 6–8-week-old *Cdx2-Cre*^+^/*Apc*^F/+^ mice (*n* = 14, **e**) or *Cdx2-Cre-ERT2*^*+*^/*Apc*^F/F^ (*n* = 8, **f**) mice. **e** Bone marrow recipient mice were sacrificed at 5 months of age, and their MLN and tumors were analyzed by flow cytometry. **f** Bone marrow recipient mice further received tamoxifen injection two months following bone marrow transfer and were sacrificed one month later. MLN and tumor tissues were harvested from these mice and analyzed by flow cytometry. *p < 0.05.

During bacterial infection, GSDMD activation results in pore formation on the plasma membrane, and the release of IL-1 family cytokines, which subsequently recruit myeloid cells and elicits inflammation [39]. Consistent with this notion, loss of GSDMD in mouse tumors resulted in reduced numbers of Gr-1^+^ CD11b^+^ immature myeloid cells (Fig. 3a). Reduction in the percentage of Gr-1^+^ CD11b^+^ immature myeloid cells in *Gsdmd*^−/−^ tumors is accompanied by the increase in F4/80^+^ macrophages (Fig. 3b). Immature myeloid cells have been shown to suppress the activity of anti-tumor T cells, and are designated as myeloid-derived suppressor cells (MDSCs) [40]. We also found reduced arginase 1 (Arg1) and COX2 expression in colorectal tumors that lack GSDMD, supporting the notion that GSDMD mediates the recruitment of MDSCs to tumors (Fig. 3c). GSDMD is dispensable for the accumulation of CD4^+^ T cells in tumors (Fig. 3d). In addition, ablation of GSDMD in the mouse model of sporadic colon cancer also did not alter the frequency of Th17 cells or regulatory T cells (Tregs) (Fig. 3d). The recruitment of Gr-1^+^ CD11b^+^ cells is mediated by the function of GSDMD in non-hematopoietic cells, as ablation of GSDMD in blood cells did not alter the abundance of these cells both in sporadic and inducible CRC mice (Fig. 3e, f).

### NLRP3 inflammasome pathway activates GSDMD in colon cancer

**Fig. 4:**
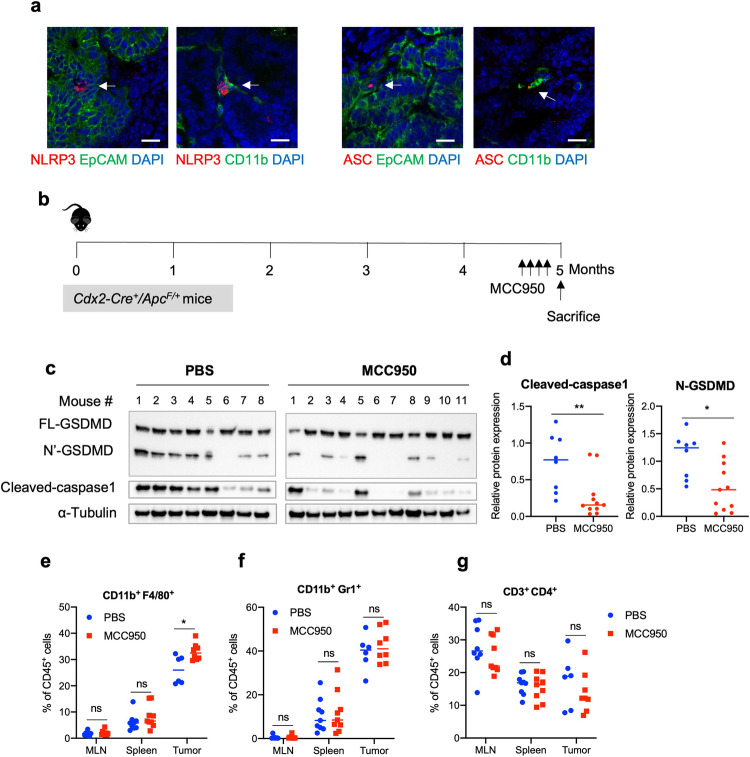
NLRP3 induces the activation of GSDMD in colorectal tumors. **a** 5-month-old *Cdx2-Cre*^*+*^ *Apc*^F/+^ mice were sacrificed, and their colorectal tumors were cryosectioned, followed by immunostaining of indicated antibodies and analysis by confocal microscopy. Representative pictures of 5 mice were shown. Scale bars = 20 μm. **b**–**g** 5-month-old *Cdx2-Cre*^*+*^/*Apc*^F/+^ mice bearing colorectal tumors were given *i.p*. injection of NLRP3 inhibitor MCC950 (10 mg/kg) once every 2 days for 7 days. Mice were sacrificed 1 day after the last dose of MCC950 injection, and their mesenteric lymph nodes (MLN), spleen, and colorectal tumors were harvested for analyses. **c** Tumors from the mice injected with MCC950 or PBS were dissected and analyzed by Western blotting. Each lane represents the pooled tumor lysate of one tumor-bearing mouse. **d** Quantified levels of cleaved caspase 1 and N’-GSDMD in tumors. PBS: *n* = 8, MCC950: *n* = 11. **e**–**g** Cells from mouse MLN, spleen, and tumors were dissociated, stained with fluorescent-conjugated antibodies and a live/dead dye, and analyzed by flow cytometry (*n* = 6). *p < 0.05 and **p < 0.01.

Several inflammasome pathways have been implicated in cancer development. Among them, the NLRP3 pathway has been shown to activate GSDMD in a mouse model of colitis-associated colon cancer and mediate the release of IL-1β and IL-18 [25, 30, 41]. Therefore, we tested if NLRP3 inflammasomes are activated in sporadic colon cancer. Indeed, immunostaining of mouse colonic tumors shows that NLRP3 forms specks in transformed epithelial cells, and less frequently, in tumor-infiltrating CD11b^+^ myeloid cells (Fig. 4a). Formation of NLRP3 specks in cells indicates their aggregation and activation [11]. Also, we observed speck formation by the inflammasome adaptor protein ASC (Fig. 4a). To test the role of NLRP3 inflammasome in colon cancer, we treated mice bearing established colorectal tumors with an inhibitor of NLRP3 (MCC950) (Fig. 4b). Inhibition of NLRP3 resulted in reduced cleavage and activation of caspase 1 and its downstream effector protein GSDMD (Fig. 4c, d). NLRP3 inhibition also resulted in increased levels of IL-1 family cytokine mRNAs, and the accumulation of the pro-form of these cytokines (Supplementary Fig. 1). In addition, NLRP3 inhibition also increased the population of F4/80^+^ macrophages in colorectal tumors, similar to what was observed in GSDMD knockout mice (Fig. 4e). We did not observe a significant change in the proportion of Gr-1^+^ myeloid cells or T cells upon NLRP3 inhibition (Fig. 4f, g). These data indicate that NLRP3 activates caspase 1 and GSDMD in tumor cells. Activated caspase 1 also cleaves and activates IL-1 family cytokines, which have been shown to promote tumor-associated inflammation. Consistently, ablation of GSDMD in colorectal tumors did not result in any change in the activation of caspase 1, or IL-1 family cytokines or formation of NLRP3 or ASC specks (Supplementary Fig. 2).

### Gut bacteria activate GSDMD in early-stage CRC

**Fig. 5:**
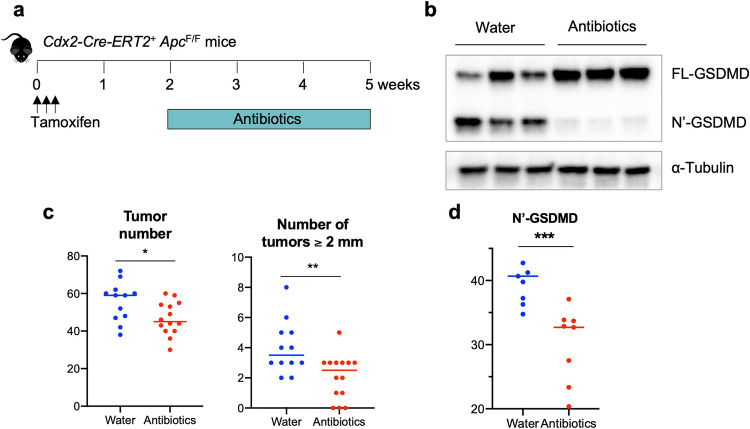
Commensal bacteria activate GSDMD in early-stage CRC. 2-month-old *Cdx2-Cre-ERT2*^*+*^/*Apc*^F/F^ mice were given *i.p*. injection of tamoxifen (75 mg/kg) daily for 3 consecutive days. 2 weeks after the last dose of tamoxifen, mice were treated with a cocktail of antibiotics in drinking water (100 μg/ml neomycin, 50 μg/ml vancomycin, 50 μg/ml imipenem, 100 μg/ml metronidazole, 50 μg/ml streptomycin and 100 units/ml penicillin) for three weeks. Fresh antibiotics were given every week. **a** Scheme of tamoxifen and antibiotic treatment. **b** Representative Western blotting of colorectal tumors from mice treated by normal drinking water or antibiotics. **c** Quantified levels of cleaved (N’-terminal) GSDMD in tumors. *n* = 7 for water, and *n* = 9 for antibiotics treated mice. **d** Mice were sacrificed at the end of 3-week antibiotic treatment, and their colorectal tumors were measured by a caliper. Total number of tumors (left panel) and the number of tumors with a diameter equal or larger than 2 mm (right panel) are shown (*n* = 12). *p < 0.05, **p < 0.01, ***p < 0.001.

Ablation of GSDMD in our mouse model of sporadic CRC resulted in reduced tumor numbers, but not tumor size (Fig. 2a), suggesting that GSDMD may mainly promote the early phases of CRC, i.e., tumor initiation/promotion, but not tumor growth. Next, we wanted to understand the mechanism by which the inflammasome-GSDMD axis is activated in CRC. Previous work has shown that transformed epithelial cells lose barrier function, allowing the infiltration of gut bacteria and their degradative products into tumor stroma [6]. We hypothesized that the activation of GSDMD is also dependent on commensal bacteria, and this would occur during early-stage CRC development. To test this idea, we treated early-stage CRC mice with a cocktail of antibiotics to deplete their gut bacteria (Fig. 5a). *Cdx2-Cre-ERT2*^*+*^*/Apc*^F/F^ mice were given 3 consecutive daily doses of tamoxifen to induce the ablation of the *Apc* tumor suppressor gene in colorectal epithelial cells. Two weeks after tamoxifen-induced *Apc* ablation, mice were treated with an antibiotic cocktail in their drinking water for another 3 weeks. This treatment scheme reduces gut bacterial load by 10,000-fold [6]. Compared to control mice, antibiotic-treated mice showed significantly reduced GSDMD cleavage, and accumulation of full-length GSDMD in these early tumors (Fig. 5b, c). Depletion of gut bacteria in the 3-week period of antibiotic treatment also reduced the number of visible tumors in the colon, especially those with diameters larger than 2 mm (Fig. 5d). Overall, these data indicate that commensal bacteria promote the activation of GSDMD and development of early-stage CRC.

### GSDMD activation turns on the ESCRT machinery in CRC tumors

**Fig. 6:**
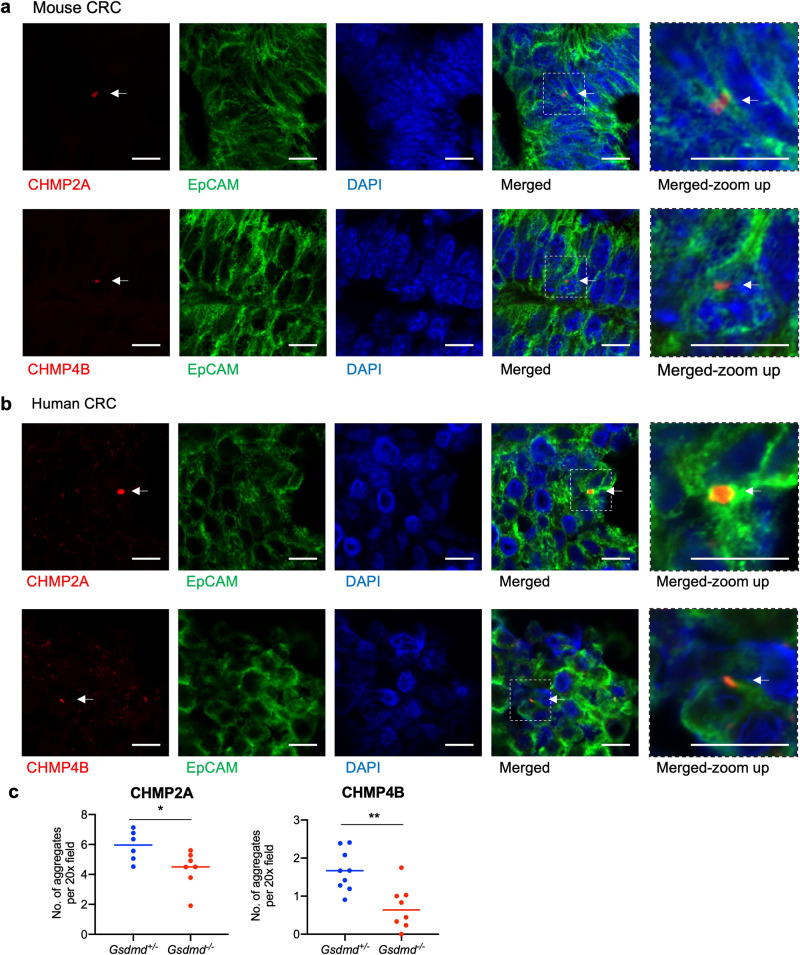
GSDMD activates the ESCRT complex of membrane repair in human and mouse CRC. **a**–**c** 5-month-old *Cdx2-Cre*^*+*^/*Apc*^F/+^ mice that harbor heterozygous (*Gsdmd*^+/−^) or null (*Gsdmd*^−/−^) alleles of GSDMD were sacrificed. Their colorectal tumors were cryosectioned and stained for indicated markers. **a** Representative staining of *Gsdmd*^+/−^/*Cdx2-Cre*^*+*^/*Apc*^F/+^ tumors. **b** Human colorectal adenocarcinoma specimens were cryosectioned and stained with indicated markers. Representative images from 4 specimens are shown. **c** Numbers of CHMP2A (*n* = 6) and CHMP4B (*n* = 9) aggregates per field were analyzed by immunostaining of these markers on cryosectioned mouse tumors. Scale bars = 10 μm. *p < 0.05 and **p < 0.01.

Activated GSDMD forms pores on the plasma membrane through the oligomerization of its N-terminal fragment, leading to the secretion of processed IL-1β and IL-18, and the loss of membrane potential. Formation of GSDMD pores may also lead to the activation of the Ca^2+^-dependent ESCRT membrane repair machinery, resulting in the prevention of pyroptosis despite the formation of GSDMD pores [21]. ESCRT-mediated membrane repair also assists cancer cells’ resistance to perforin-mediated T-cell attack [42]. We hypothesize that ESCRT actively removes GSDMD pores in colorectal cancer cells to prevent their cell death, thus allowing the tumor-promoting function of GSDMD. We observed selective activation of the ESCRT-III effector proteins CHMP2A and CHMP4B in both human and mouse CRC tumor cells (Fig. 6a, b). These CHMP protein aggregates colocalize with the plasma membrane protein EpCAM, suggesting the presence of membrane repair (Fig. 6a, b). Ablation of GSDMD reduced the number of tumor cells that harbor aggregated CHMP2A and CHMP4B (Fig. 6c). These data suggest that cleavage and activation of GSDMD in tumor cells leads to the formation of pores on the tumor cell membrane and subsequent activation of the ESCRT complex, potentially initiating membrane repair.

## Discussion

Activation of GSDMD leads to inflammatory cell death [12, 18, 31]. Further, *Gsdmd*^−/−^ mice were shown to develop more tumors in the gut when subjected to the AOM/DSS model of CAC induction [24]. This led to our initial expectation that GSDMD should kill cancer cells when activated, and thus serve as a tumor-suppressing factor. To our surprise, ablation of GSDMD resulted in reduced tumor numbers and tumor load, suggesting a tumor-promoting role in sporadic CRC, which accounts for a majority of CRC in humans. This pro-tumor function may be attributed to the release of inflammatory cytokines, such as IL-1β, following GSDMD pore formation. Based on our observations, we speculate that IL-1β, following inflammasome activation and GSDMD pore formation, attracts myeloid cells to the tumor and promotes CRC development by signaling to cancer cells, myeloid cells, as well as tumor-infiltrating T cells [43]. IL-1β signaling on T cells promotes the production of IL-17, which may in turn attract immature myeloid cells to the tumor [43]. These immature myeloid cells have been shown to exert immunosuppressive functions (thus termed myeloid-derived suppressor cells, MDSCs) by secreting various cytokines and extracellular enzymes such as VEGF, TGFβ, ARG1, and IL-10, thus inhibiting T cells, NK cells, and dendritic cells, while promoting Treg cell proliferation [44, 45].

Cleavage and activation of GSDMD were found in both epithelial-derived tumor cells and tumor-infiltrating immune cells, but the former population represents predominant site of GSDMD activation. The tumor-promoting role of GSDMD in sporadic CRC also suggests that tumor cells may be protected against GSDMD pore formation and subsequent pyroptosis. We reason that this could be due to the observed accumulation of aggregated CHMP2A and CHMP4B proteins, members of the ESCRT membrane repair machinery, in the membrane of both human and mouse CRC tumor cells, that were decreased upon ablation of GSDMD in mice. ESCRT complex responds to Ca^2+^ influx caused by GSDMD pore formation in inflammasome-activated cells and removes such pores to prevent pyroptosis [21], thus potentially implicating itself in promoting tumor progression or resistance. Blockade of Ca^2+^ influx by a Ca^2+^ chelator prevents ESCRT-mediated membrane repair and enhances GSDMD-caused pyroptosis in cancer cells [46]. It remains to be tested if ESCRT-mediated membrane repair is responsible for the tumor-promoting role of GSDMD in sporadic CRC, and if abolishing this process will revert the function of GSDMD in causing tumor cell death and tumor inhibition.

The presence of commensal bacteria in the gut is required for the activation of GSDMD and the outgrowth of early CRC tumors (Fig. 5c) [6]. Intriguingly, GSDMD is also activated in the normal colonic epithelium [47]. GSDMD has been found to drive mucus granule secretion in goblet cells, thus is required for mucus layer formation in the gut [47]. Activation of GSDMD and secretion of mucus are also dependent on gut bacteria, akin to what we found for the activation of GSDMD in CRC tumors [47]. Commensal bacteria and their products may penetrate the tumor surface and reach the tumor stroma through the defective epithelial barrier caused by gut epithelial cell transformation [6]. We found decreased levels of NLRP3 protein and activated GSDMD in large colorectal tumors compared to small ones, suggesting the inefficiency of bacteria and their products reaching the core of large colorectal tumors. Bacterial products, such as LPS, have been shown to serve as “priming” factors to enhance the expression of NLRP3 and other inflammasome pathway components [48]. NLRP3 is activated by multiple stimuli, such as uric acid crystals, extracellular ATP, toxins, as well as viral, bacterial, fungal, and protozoan pathogens [9]. One potential pathway for NLRP3 activation is the efflux of K^+^ following non-canonical inflammasome pathway-mediated GSDMD pore formation [49, 50]. However, ablation of GSDMD in sporadic colon tumors did not result in altered IL-1α, IL-1β, and IL-18 as well as NLRP3 or ASC speck formation. It is therefore likely that NLRP3 is activated through other mechanisms, such as the cellular stress caused by the chronic inflammation following bacterial invasion to the tumor microenvironment, that needs to be investigated further. Indeed, antibiotic treatment in the mouse model of inducible CRC reduced GSDMD cleavage and slowed down the development of early-stage CRC (Fig. 5). Thus, our results demonstrate a gut bacteria-driven inflammasome activation that leads to GSDMD activation and enhanced tumor development.

### Supplementary information

Supplementary Figures 1 and 2

## Data Availability

All data are available upon request made to the corresponding author.
