# Supplementary material for: Gut microbiota-mediated activation of GSDMD ignites colorectal tumorigenesis
Source: Cancer Gene Ther. 2024 Jun 19;31(7):1007–17. doi: 10.1038/s41417-024-00796-2 (PMC11257976; doi:10.1038/s41417-024-00796-2)
Supplement: Supplementary file 1 — Supplementary Figures 1 and 2 [file 41417_2024_796_MOESM1_ESM.pdf]

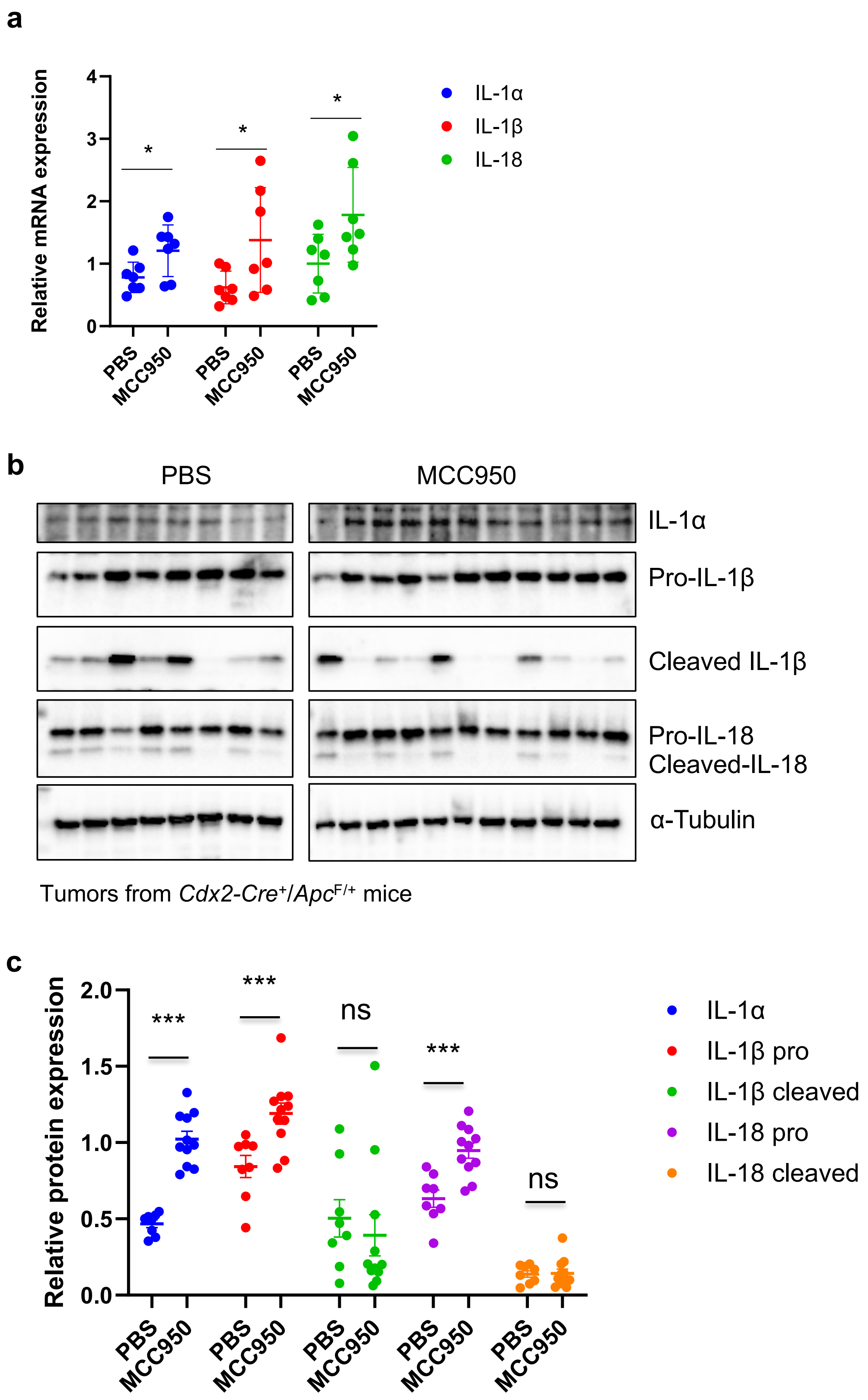
a
Relative mRNA expression
IL-1α
IL-1β
IL-18
PBS
MCC950
b
PBS
MCC950
IL-1α
Pro-IL-1β
Cleaved IL-1β
Pro-IL-18
Cleaved-IL-18
α-Tubulin
Tumors from Cdx2-Cre+/ApcF/+ mice
c
Relative protein expression
***
ns
IL-1α
IL-1β pro
IL-1β cleaved
IL-18 pro
IL-18 cleaved
PBS
MCC950

Supplementary Figure 1: Level of IL-1 family cytokines following NLRP3 inhibition. 5-month-old *Cdx2-Cre*$^{+}$/*Apc*$^{F/+}$ mice bearing colorectal tumors were given *i.p.* injection of NLRP3 inhibitor MCC950 (10mg/kg) once every 2 days for 7 days. Mice were sacrificed 1 day after the last dose of MCC950 injection, and their colorectal tumors were harvested for analyses. **a**: Relative mRNA expression of IL-1α, IL-1β or IL-18 in colorectal tumors from PBS or MCC950 injected mice (n=7). GAPDH was used as an endogenous control. **b**: Tumors from the mice injected with MCC950 or PBS were dissected and analyzed by Western blotting. Each lane represents the pooled tumor lysate of one tumor-bearing mouse. **c**: Quantified levels of IL-1α, IL-1β (pro and cleaved) or IL-18 (pro and cleaved) in tumors. PBS: n=8, MCC950: n=11. Data are represented as mean +/- SD. P values: ns = not significant, * p<0.05, ***p<0.001.

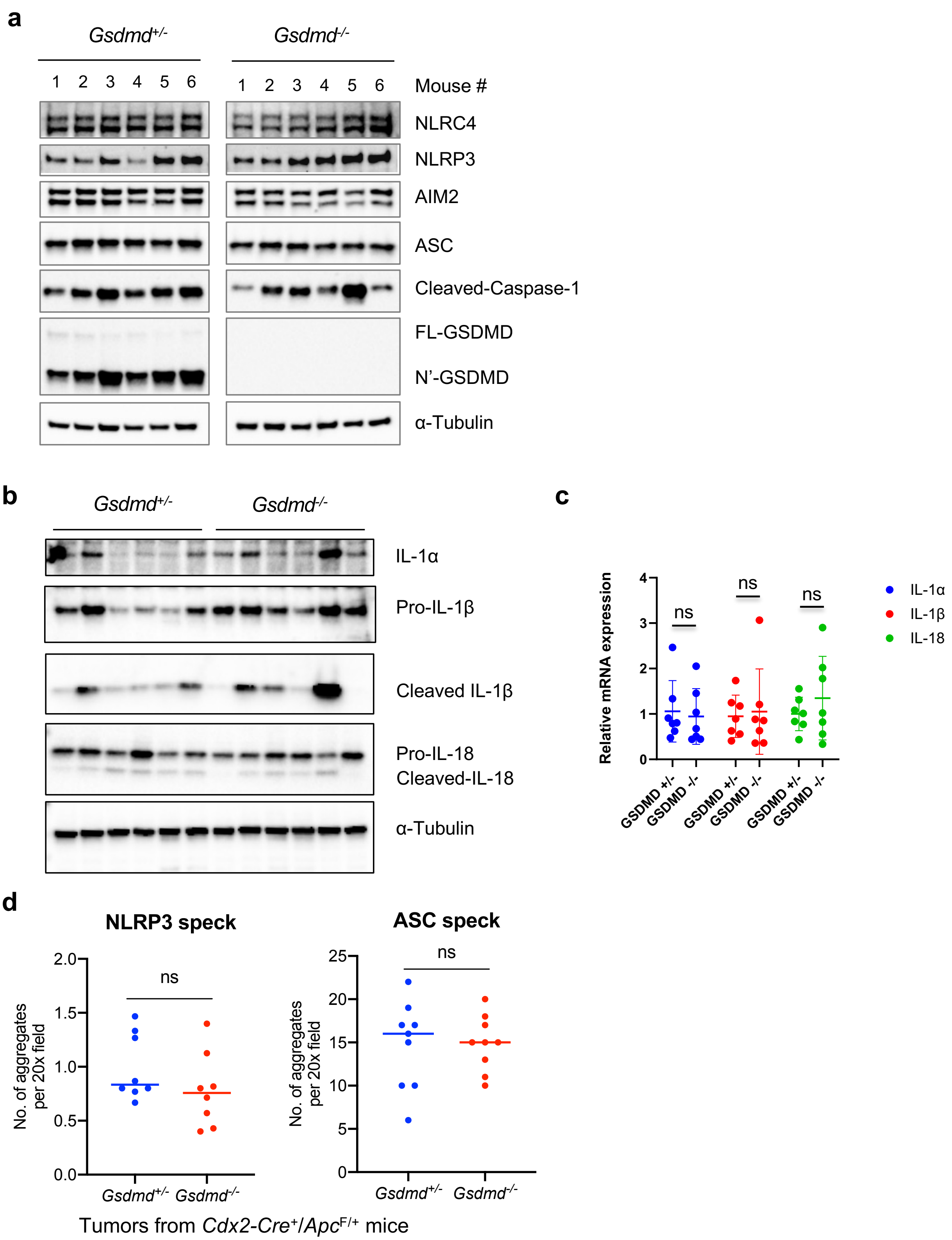

Supplementary Figure 2: GSDMD does not feedback regulate the NLRP3 pathway. **a**: 5-

month-old *Cdx2-Cre*$^{+}$/*Apc*$^{F/+}$ mice that harbor heterozygous (*Gsdmd*$^{+/-}$, as controls) or null (*Gsdmd*$^{-/-}$) alleles of GSDMD were sacrificed and subjected to Western blotting analysis for indicated inflammasome signaling molecules. **b**: 5-month-old *Cdx2-Cre*$^{+}$/*Apc*$^{F/+}$ mice that harbor heterozygous (*Gsdmd*$^{+/-}$, as controls) or null (*Gsdmd*$^{-/-}$) alleles of GSDMD were sacrificed and subjected to Western blotting analysis for indicated proteins. **c**: Relative mRNA expression of IL-1α, IL-1β or IL-18 in colorectal tumors from *Cdx2-Cre*$^{+}$/*Apc*$^{F/+}$ mice that harbor heterozygous (*Gsdmd*$^{+/-}$, as controls) or null (*Gsdmd*$^{-/-}$) alleles of GSDMD were analyzed by RT-qPCR (n=7). GAPDH was used as an endogenous control. **d**: Images of immunostaining were obtained from tumors harboring heterozygous (*Gsdmd*$^{+/-}$) or null (*Gsdmd*$^{-/-}$) alleles of GSDMD by confocal microscopy, and were analyzed for the numbers of NLRP3 (n=8) and ASC (n=8) aggregates per field. Data represented as mean +/- SD. P values: ns = not significant.
